# Development of Novel Drugs from Marine Surface Associated Microorganisms

**DOI:** 10.3390/md8030438

**Published:** 2010-03-01

**Authors:** Anahit Penesyan, Staffan Kjelleberg, Suhelen Egan

**Affiliations:** School of Biotechnology and Biomolecular Sciences and Centre for Marine Bio-Innovation, University of New South Wales, Sydney 2052, Australia; E-Mails: A.Penesyan@unsw.edu.au (A.P.); S.Kjelleberg@unsw.edu.au (S.K.)

**Keywords:** marine epibiotic microoorganisms, bioactive, antimicrobial, natural products

## Abstract

While the oceans cover more than 70% of the Earth’s surface, marine derived microbial natural products have been largely unexplored. The marine environment is a habitat for many unique microorganisms, which produce biologically active compounds (“bioactives”) to adapt to particular environmental conditions. For example, marine surface associated microorganisms have proven to be a rich source for novel bioactives because of the necessity to evolve allelochemicals capable of protecting the producer from the fierce competition that exists between microorganisms on the surfaces of marine eukaryotes. Chemically driven interactions are also important for the establishment of cross-relationships between microbes and their eukaryotic hosts, in which organisms producing antimicrobial compounds (“antimicrobials”), may protect the host surface against over colonisation in return for a nutrient rich environment. As is the case for bioactive discovery in general, progress in the detection and characterization of marine microbial bioactives has been limited by a number of obstacles, such as unsuitable culture conditions, laborious purification processes, and a lack of de-replication. However many of these limitations are now being overcome due to improved microbial cultivation techniques, microbial (meta-) genomic analysis and novel sensitive analytical tools for structural elucidation. Here we discuss how these technical advances, together with a better understanding of microbial and chemical ecology, will inevitably translate into an increase in the discovery and development of novel drugs from marine microbial sources in the future.

## 1. Introduction

The number of natural products, discovered from various living organisms including plants, animals and microbes, to date exceeds 1 million [1], with the majority (40–60%) derived from terrestrial plants. Of these natural products, 20–25% possess various bioactive properties including antibacterial, antifungal, antiprotozoal, antinematode, anticancer, antiviral and anti-inflammatory activities.

Plants and plant extracts have been used for the treatment of human diseases for millennia, and their use has been recorded in the most ancient archaeological sources. In contrast, the exploration of microorganisms as producers of therapeutical agents only began in the 20th century [2]. However, despite this relatively short history, nearly 10% of all currently known biologically active natural products are of microbial origin. These include the majority of antibiotics, clearly demonstrating the potential of microorganisms as an emerging source for the production of biologically active products. Indeed, by the 20th century microbially derived bioactives had become the foundation of modern pharmaceuticals [3]. For example, the production of antimicrobials is observed in 30–80% of actinomycete and fungal strains screened in various studies [4,5]. Moreover, mathematical models predict that the number of undiscovered antibiotics from actinomycetes could be in the order of 10^7^ [6].

An emerging source of new bioactives may result from the many recent studies of microbial diversity in the marine environment, particularly those microbes associated with marine plants and animals [7–16]. Several studies have demonstrated that “living surfaces” represent an environment rich in epibiotic microorgansims that produce bioactives [17–20]. Nevertheless, the vast biotechnological potential of marine epibiotic microorgansims remains mostly unexplored [21]. This review discusses the importance of exploring new sources potentially rich in bioactives, and highlights the significance of considering the chemical ecology of marine microorganism-host associations for the targeted isolation of bioactive producing microorganisms.

## 2. Exploring the Under-Explored–Marine Microorganisms as a Source of New Drugs

In the past, it was presumed that the marine environment was a “desert” with scarcity of life forms [22]. However, it is now clear that the oceans are thriving with tremendous diversity of living microorganisms, with cell counts of 10^6^–10^9^ cells per milliliter [7,8] and levels of species diversity and richness predicted to exceed many of the Earths rainforests [23–26]. This microbial diversity is presumed to translate into metabolic diversity resulting in the potential for new bioactives to be discovered. Indeed, in the past decades we have witnessed an increase in the number of marine natural products a large proportion of which are of microbial origin [27] (MarinLit database, University of Canterbury: http://www.chem.canterbury.ac.nz/marinlit/marinlit.shtml). For example, in 2007 there was a significant increase (38%), compared to the preceding year, in the number of new marine microbially derived compounds [27]. In addition to structural variety, bioactives obtained from marine microorganisms are known for their broad range of biological effects, which include antimicrobial, antiprotozoan, antiparasitic, and antitumour activities [28–35], as well as antifouling activities that prevent the surface-settlement of various marine organisms [36–38]. Many of these compounds are noted for their high potency, which could be related to the need to overcome the dilution of allelochemicals in the seawater [26,39].

Use of bioactive producing marine eukaryotes in large-scale production faces many difficulties mainly due to the fact, that, in many cases, the eukaryotic organism is killed in the process of obtaining the bioactive, and many of these eukaryotes cannot be cultured in laboratory, but need to be hand-picked by SCUBA [40]. It also raises the issue of sustainability of these organisms in the nature. In contrast, many bioactive producing marine microorganisms can be easily cultured and manipulated in bioreactors and, therefore, represent the best renewable source of biologically active compounds [41].

### 2.1. Marine Surface Associated Microoorganisms

The marine environment is a complex ecosystem with an enormous diversity of different life forms often existing in close associations. Among these, microorganism-eukaryote associations have gained significant attention in the past decade [21].

The surfaces of all marine eukaryotes are covered with microbes that live attached in diverse communities often embedded in a matrix, forming a biofilm. The microbial consortia living on various eukaryotes differ significantly from each other and from the microorganisms living in the surrounding seawater [9–16,42,43]. For example, a DGGE based comparison of the microbial community composition of the coral *Montastraea franksi* and the surrounding seawater revealed almost no overlap [42]. Likewise, Longford *et al.*, [10] found only two (out of a total of one hundred) bacterial species that were common to three different marine sessile eukaryotes. Moreover, host specificity has also been illustrated by studies that have shown the presence of unique stable communities living on geographically distant individuals belonging to the same species [14,44].

In contrast to free living planktonic microorganisms, which often encounter fluctuations in environmental conditions, requiring quick short-term adaptive responses, surface associated microorganisms supposedly have developed more specialised and stable adaptations, specific to the microenvironment created by a particular host. The high level of specificity of microbial communities on various marine eukaryotes highlights the existence of close cross-relationships between microbial epibionts and their eukaryotic hosts. In fact, some epibiotic microorganisms have been shown to be essential for the normal life and development of the eukaryote, for example, being involved in the development of host morphology [45–49]. Host specific bacteria have also been shown to be vertically transferred from the parental eukaryotic organism to its offspring, which indicates the importance of these bacteria for the host. Such inheritance of members of the microbial community has been reported to occur in sponges [16,50,51], bivalves [52] and ascidians [53]. While the exact nature of the relationship between the microorganisms and their hosts remains unclear, it has been hypothesized that the microbial partners construct chemical microenvironments with the eukaryotic host and live in syntrophy, participating in cycling of nutrients, as well as preventing predation of the host via the production of bioactive molecules [50].

Marine surface associated bacteria are often metabolically linked with their host. For example, epiphytes belonging to the *Roseobacter* lineage are known to degrade the algal osmolyte dimethylsulfoniopropionate (DMSP) yielding the climate-relevant gas dimethylsulfide (DMS) and are regarded as major players in sulphur cycling in the ocean [54,55]. In contrast, some marine eukaryotes heavily rely on the metabolites produced by their microbial symbionts to survive. For example, some marine sponges use the carbon produced by their associated photosynthetic cyanobacteria [56] and may even rely on their autotrophic cyanobacterial symbionts to provide more than 50% of their energy requirements, which allows them to grow in low-nutrient environments [57].

Close metabolic associations between microorganisms and their host can make it difficult to reveal which partner organism is responsible for the production of a particular metabolite. As a result, many bioactive products, previously ascribed to the eukaryotes, have later been found to be produced by associated microorganisms [58–66]. For example, the microbial origin of the cytotoxic compound bryostatin was demonstrated by the identification of polyketide synthase genes, involved in its biosynthesis, in the genome of the bryozoan bacterial symbiont “*Candidatus* Endobugula sertula” [67]. Moreover, it was proposed that microbially derived bryostatin, found on the larvae of bryozoan *Bugula neritina*, serves to defend the larvae against potential predators [68].

The interactions between the epibiotic microorganisms and their host, in which microorganisms are thought to acquire nutrients from the eukaryote, while the host benefits from the wide range of bioactives produced by its associated microorganism, seems to be widespread in the marine environment [69]. For example, the gamma-proteobacterium *Pseudoalteromonas tunicata*, known for the production of several bioactive compounds, is proposed to play a role in defending the host against surface colonisation by producing antimicrobial, antilarval and antiprotozoan compounds [70–73]. Likewise, the surfaces of the healthy embryos of the lobster *Homarus americanus* are covered almost exclusively by a single gram-negative bacterium, that produces an antifungal compound highly effective against the fungus *Lagenidium callinectes*, a common pathogen of many crustaceans [74].

The production of antimicrobials by epiphytic microorganisms could also give the producers a distinct advantage in competition with other surface-dwelling microbes. This is especially important given the fierce competition that exists on the surfaces of marine living organisms, that are relatively rich in nutrients compared to seawater, and, therefore, are attractive for numerous microorganisms [21].

The fact that many marine microorganism-host associations are based on metabolic or chemical interactions may explain the abundance of bioactive producing bacteria on living surfaces [17–19,75,76]. Thus, a better understanding of the ecological challenges and the underlying mechanisms involved in such interactions should accelerate the search for novel bioactives.

## 3. The Challenges of Microbial Bioactive Natural Product Development from Marine Epibiotic Microorganisms

**Figure 1:**
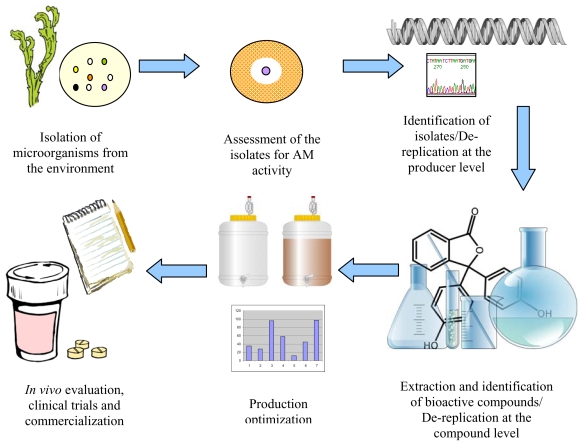
General procedure for the discovery of biologically active natural compounds, such as antimicrobials, of microbial origin. The procedure starts with the isolation of microorganisms from the environment, for example, from the surfaces of marine eukaryotes, followed by their antimicrobial activity screening and the identification of the producer organism. The bioactive compound is then purified and the chemical structure elucidated. Production optimization can be performed to maximize the yield of the desired compound for further *in vivo* trials and product development. Clip art images provided by Open Clip Art Library (www.openclipart.org) are used in the figure.

The general procedure for the isolation of natural products from marine epibiotic microorganisms includes several essential steps. The process begins with the isolation of microorganisms from the environment. Often, in the past, the isolation of microorganisms has been a random process. However there is now a growing recognition that the source of microbial samples can be important for increasing the success rate of bioactive discovery [17,20]. Thus, as discussed above, due to the various and often chemically mediated interactions that occur between microorganisms and their host and between members of the epibiotic community, isolation of microorganisms from marine living surfaces can significantly increase the chances of obtaining bioactive producing strains. After growing the microorganisms in the laboratory on nutritional media, the screening of individual isolates for biological activity is performed, for example, based on the inhibition of growth of microorganisms surrounding the test organism in the case of antimicrobials. The phylogenetic and phenotypic identification of the bioactive producing organism is then performed as the first de-replication to ensure that the organism has not been previously used for the particular activity and, subsequently, to maximise the possibility of finding a novel bioactive compound. The extraction and purification of biologically active compounds are then performed, followed by chemical structure elucidation. At this stage a second de-replication can be done to exclude already known compounds. Once novel compounds are identified, the various growth conditions of the producer organism can be assessed to optimize their production. Finally, compounds are assessed for use in the treatment of certain diseases [77,78] and in a variety of industrial settings (Figure 1).

### 3.1. Improving the Culturability and Production of Bioactives from Marine Microorganisms

The limited ability to culture the majority of environmental strains represents a major bottleneck in classical culture-based screening programs for microbial derived bioactives, including those from marine surfaces. It is estimated that the majority (98–99%) of microorganisms cannot be cultured by traditional techniques [79–81]. Nevertheless, marine living surfaces may provide an advantage as, in some cases, a higher percentage of eukaryote associated microorganisms can be readily cultured [82].

Being able to grow the organism *in vitro* provides great advantages, such as better access to its physiology [83,84]. This may allow the manipulation of different growth parameters to achieve the maximum yield of various products and for their large-scale production via fermentation. Among strategies to improve the culturability of microorganisms, those that attempt to grow the organisms under conditions that mimic the physical and chemical parameters of their natural environment, have been the most successful. For example, by using specialised environmental chambers, Kaeberlein *et al.*, [85] could successfully culture up to 40% of of all microbial cells present in a marine environmental sample.

The close associations often present between marine eukaryotic organisms and their microbial epibionts, clearly impose conditions that are difficult to replicate with standard laboratory procedures. It has, therefore, been proposed that the development of suitable culturing techniques for such organisms should involve conditions that reflect the microenvironment created by their host [86]. This approach has proven successful for the isolation of the sponge associated bacterium *Oscillatoria spongeliae* [87], for which hyperosmotic medium, resembling the osmolarity of the sponge mesophyle, was used to cultivate the organism.

Cultivation conditions, such as temperature, aeration, pH of the media, incubation time and media composition, can affect the production of the desired metabolite, and, therefore, must be taken into account and fine-tuned [88–94]. Usually this requires a producer strain to be grown in the conditions optimal for the production of the active compound. These conditions can differ significantly from the optimal growth conditions of the strain. In some cases, the producer organism is grown under a variety of conditions in parallel and the differences in metabolic spectra are assessed [95–97]. Marine surface associated microorganisms may also require conditions that resemble their native environment in order to produce the maximum amount of bioactives. For example, several studies have shown an increase in the production of antimicrobial compounds when the surface associated bacteria were grown, *in vitro*, to form surface attached biofilms [41,98,99]. In addition, Okazaki *et al.*, [100] have shown that marine isolate SS-228 was able to produce the antibiotic compound only when the growth medium was supplemented with powdered *Laminaria* seaweeds, common in the habitat from which strain SS-228 was obtained. It is now becoming clear that knowledge of a microorgansims’ natural habitat, including the specifics of the host organism, can improve production of microbial derived bioactives.

Sequence information obtained via the sequencing of the environmental DNA (“metagenome”) can greatly assist in understanding the metabolic potential of the organisms present in the environment, and thus guide the development of specialised cultivation conditions. For example Tyson *et al.*, [101] successfully cultured *Leptospirillum ferrodiazotrophum* by developing a selective isolation strategy. The predicted nitrogen fixing capability of this organism, based on the sequence information of an acid mine drainage biofilm community, underlined the development of that strategy.

Over the past decade genomics has emerged as an alternative to directly culturing microorganisms for the isolation of new bioactives. In particular, functional metagenomics was first developed to tackle the biotechnological potential of unculturable microorganisms. In this approach the DNA obtained from the environment (“environmental DNA”) is inserted into a host organism, such as *E. coli*, and a functional screen of libraries is performed to detect the desired activity in the clones [102–107]. Some of the successes of this approach were the discovery of terragine A [108], bioactive *N*-acyl-tyrosine derivatives [109] as well as indirubin [110] from the soil metagenomes.

Functional (meta-)genomics can provide an insight into the genes and gene clusters involved in the production of certain metabolites, and, thus, provide information about the possible biosynthetic pathway leading to that metabolite [111]. Such an approach has been used by Burke *et al.*, [112] to propose the biosynthetic pathway of the antifungal compound tambjamine produced by the marine bacterium *P. tunicata*. Recently the same approach was successful in identifying two positive clones from a *P. tunicata* genome library, with different modes of action against the nematode *Caenorhabditis elegans* (Ballestriero *et al.*, unpublished data). In addition, a functional (meta-) genomics approach provides an advantage for further purification of the bioactive compound produced by a clone, since the “extra” metabolite can be relatively easily pinpointed by using a reference non-bioactive-producing clone [113].

However, despite some success, currently the hit rate of using metagenomic functional screening to obtain bioactive producing clones generally remains low, in the order of 1 in 10,000 [109,114], or even as low as 1 in 730,000 [115] clones screened. This low hit rate is mainly a result of the limited ability of host expression strains to express compounds of foreign origin [78,116,117]. Therefore it is possible that with improvements of such strains the hit rate for positive clones in metagenomics functional screens will greatly increase. For example, recent studies have demonstrated that the use of a variety of host expression strains can assist in the expression of the desired metabolite [118–121]. Specifically, these strains are often chosen based on possible similarities with the producer bacteria (if known), such as, for example, the similarities in codon usage, as well as presence of specific machinery necessary for the production of particular metabolites [119,120].

Alternatively, shotgun sequencing of environmental DNA and subsequent data analysis has the potential to identify genes encoding new structures of known compound classes, e.g., polyketide synthases (PKS) and non-ribosomal peptide synthetases (NRPS) usually involved in production of bioactive secondary metabolites [122]. For example, recent analysis of the genome of *P. tunicata* has revealed the presence of nine NRPS [123]. Two products of these NRPS with predicted biological activity have been recently identified in the laboratory via heterologous expression, and their presence was confirmed in the original strain of *P. tunicata* by varying its conditions of growth [124].

Moreover, the availability of sequence data from a variety of microorganisms has further highlighted the importance of developing culturing conditions that would be suitable for the production of bioactives. There are now several examples of genes involved in the biosynthesis of bioactives found in non-bioactive producing organisms, suggesting that, given suitable growth conditions, these organisms have the potential to produce bioactive metabolites. For example, the genome of the myxobacterium *Stigmatella aurantiaca* DW4/3-1 showed the presence of multiple polyketide synthase/non-ribosomal peptide synthetase gene clusters, which had not been previously observed in this organism [125]. In another example, the previously unknown potential of the production of the antitumour compound terrequinone A in the fungus *Aspergillus nidulans* was revealed [126].

### 3.2. De-Replication

In the early years of antibiotic discovery the selection of antibiotic producer strains was based on morphology rather than genotypes, resulting in redundancy in many natural product extract libraries [127,128]. The initial success in the discovery of many antibiotic compounds from natural sources included thousands of compounds being described within a few decades. However, the lack of a systematic approach often resulted in the frequent re-discovery of known compounds. Therefore, it is important to put considerable effort into the de-replication process for early detection of both the known producer organisms as well as the known bioactive compounds.

Currently, 16S rRNA gene sequencing [129] and phenotypic characterisation can serve for the identification of producer organisms, and, hence, may reveal whether a given microorganism or its close relatives have been previously known to produce certain bioactives, in order to focus the efforts on organisms with yet uncharacterised activity.

Concerning the early detection of known bioactive compounds, advances in the development of new chemical analysis techniques, coupled with a database evaluation, can serve as tools for rapid detection of known compounds requiring only small quantities of sample and/or minimal efforts in sample preparation [130–132]. Hence, they may greatly assist in preventing the waste of resources, which would otherwise be necessary for scale-up and characterisation of the bioactive compounds.

The exploration of relatively unexplored environments can also assist in finding novel microorganisms and chemical structures, and, hence, minimise the re-discovery of known compounds. The oceans have proven to be a habitat for many unique microbes [133], such as, for example, the recently discovered marine genera *Salinispora* and *Marinophilus* [133,134]. In addition, some of the members of these groups were found to produce structurally novel bioactive metabolites, for example, salinosporamides, a family of compounds with cytotoxic activity, were successfully isolated from *Salinnispora tropica* [135]. Likewise, the structurally novel compounds marineosins [136] and largazole [137] have been recently isolated from marine bacteria belonging to the actinomycete and cyanobacterial groups respectively. Recently discovered marine bioactive natural products including compounds with unique structures are reviewed in [138]. This supports the idea that the unique chemical and physical parameters of the marine environment can lead to the evolution of life forms that could also produce metabolites with novel chemical scaffolds [139,140].

### 3.3. Purification of Bioactives from Crude Extracts

Despite their potential, full characterisation of marine microbially derived bioactives, as well as the development of extraction and purification strategies can be a long and laborious process requiring a great deal of manual work, with little room for automation [78,141].

Purification and structure elucidation of mass limited sample material is considered a major bottleneck. This is usually because the compound of interest often represents less than 1% of the crude extract, which, in most cases, is a mixture of hundreds of different compounds. Therefore, every extract has its unique combination of “contaminants” necessitating a specific approach; as a result, development of the purification strategies remains largely experimental [142].

Furthermore, obtaining adequate quantities of bioactive compounds, necessary for structure elucidation and evaluation, usually requires extensive optimization of conditions and scale-up [143].

To facilitate the chemical characterisation, analytical methods are constantly being developed and improved, one of the major lines of improvement being the possibility of using small quantities of sample and easy sample preparation. For example, the recently developed Ultra High Performance Liquid Chromatography (UPLC) coupled to high resolution mass spectrometers (MS), as well as capillary probe nuclear magnetic resonance spectrometers have greatly assisted the process of natural product discovery from mass limited samples [130,144,145]. Likewise, the newly developed Desorption Electrospray Ionisation Mass Spectrometry (DESI-MS) technique allows for the rapid detection of the compounds requiring minimal effort to be spent on sample preparation [131,132]. These techniques may soon eliminate, or, by some estimates, have already eliminated the purification and chemical characterisation step as a major drawback in natural product discovery [146–150].

## 4. Is There an Alternative to Natural Product Discovery and Development?

The challenges of natural product research have resulted in a search for an alternative to bioactive product development.

Combinatorial biosynthesis has emerged as one of the alternatives and is defined as “the application of genetic engineering to modify biosynthetic pathways to natural products in order to produce new and altered structures using nature’s biosynthetic machinery” [151]. It involves the use of genes from different biosynthetic pathways, in various combinations, in order to generate libraries of hybrid structures. However, in practice, this approach is rather problematic. Firstly, it involves the construction of various mutant organisms and, therefore, is very labour-intensive and costly. Secondly, it often relies on the low substrate specificity of enzymes in the biosynthetic pathways, which is not always the case as many enzymes are rather specific [151].

High-throughput screening (HTS) of synthetic chemical libraries is also regarded as an alternative to bioactive discovery and the development of combinatorial chemistry has allowed for smaller, more drug-like libraries to be screened against defined macromolecular targets. Furthermore, an increase in the availability of genomic data has provided more potential targets for these screens [142,152–157]. However, the first libraries of chemically synthesised compounds provided more quantity than quality; some produced more than million compounds, but were a disappointment, as they yielded very low numbers of, or no, active compounds [142,158]. For example, GlaxoSmithKline (http://www.gsk.com) have recently disclosed the results of a six-year campaign to discover broad-spectrum antibiotics that was abandoned because of the limited chemical diversity of their synthetic screening libraries [157]. These approaches obviously failed to fulfil initial expectations [159–164], and are unlikely to substitute the benefits of natural product development.

In contrast to chemical libraries, bioactives of natural product origin provide a diversity and a structural complexity with densely packed functional groups allowing maximum selectivity and interaction with the target [77,165,166]. Such complexity makes the chemical synthesis of these compounds extremely difficult [146,172–176]. Nevertheless there has been some success with the total synthesis of natural products from marine microorganisms, such as, for example, dinoflagellate toxins azaspiracids and brevetoxins, and the antibiotic compound uncialamycin produced by marine Streptomyces [167–171]. It has been suggested that the success of natural compounds is due to the fact that they have undergone natural selection and, therefore, are best suited to perform their activities [142,160,177]. Thus, further research on bioactive natural products may provide a source of new chemical structures that can guide the design of novel chemical compounds [178,179], as well as reveal yet unknown modes of action [180].

The majority of antibiotics currently used in clinical practice are of natural product origin [77,161,181]. For example, 70 out of the 90 antibiotics marketed in the years 1982–2002 originated from natural products [161]. Notably, the quinolones or fluoroqinones, one of the most successful classes of synthetic antibiotics, are also based on the structure of the natural product quinine [182]. In fact, chemical modifications based on a natural product scaffold is a widely used approach in modifying the chemical and physical properties of the molecule thus making it useful for a particular pharmacological application [183,184]. For example, Jenkins *et al.*, [185] have recently synthesised four new chemical scaffolds useful for drug development based on novel structures of a number of bioactive natural products such as the histrionicotoxins isolated from the skin of the Colombian poison dart frog, *Dendrobates histrionicus* [185]. Likewise, the chemically synthesised analogs of epothilones—compounds produced by myxobacterium *Sorangium cellitlosum* [186], have shown an increased potency against tumour cells compared to the original natural product [187,188].

## 5. Conclusions

Despite the challenges, the search and development of natural products remain an indispensable and unparalleled source of biologically active compounds. Thus, research into the diversity of bioactive natural products justifies the resources invested due to the lack of equivalent alternatives in synthetic compounds [142,178]. Microorganisms are currently accepted as the best renewable source for bioactives, and the exploration of yet underexplored sources, such as the marine living-surface habitat, has a great potential to deliver novel bioactive producing microbes useful for further drug development. Moreover, a systematic approach that takes into consideration unique ecological relationships in the marine environment, such as those discussed in this review, can greatly assist in maximizing the output of obtaining novel bioactive producing organisms and, thus, may prevent the frequent re-discovery of known compounds and the waste of resources that would be necessary for largescale high-throughput screens.
